# Design and optical optimization of a 45 kW beam-down solar-thermal concentrator

**DOI:** 10.1016/j.heliyon.2024.e39381

**Published:** 2024-10-18

**Authors:** Ramón Pujol-Nadal, Luis Guerreiro, Gabriel Cardona

**Affiliations:** a Departament d'Enginyeria Industrial i Construcció, Universitat de les Illes Balears, Palma, E-07122, Spain; b Institute of Earth Sciences (ICT), University of Evora, Évora, P-7002, Portugal; c Departament de Ciències Matemàtiques i Informàtica, Universitat de les Illes Balears, Palma, E-07122, Spain

**Keywords:** Beam-down, Cassegrain, Concentrating solar power, Ray tracing, Parametric design

## Abstract

Concentrated Solar Power (CSP) technologies offer significant potential as renewable energy sources, particularly when integrated with storage systems. To address the challenges of energy transport and re-radiation losses, beam-down configurations combined with paraboloid-type dish concentrators provide a compact and efficient solution. In this study, we perform an optical analysis of a Cassegrain concentrator using the open-source software OTSunWebApp, a ray-tracing program that allows for the spectral optical analysis of solar collectors. The analysis accounts for specular scattering of the reflectors, the spectral behavior of the materials, and the angular size of the sun. The geometric design is parametrized with six degrees of freedom, while some other parameters, such as the number of mirrors and the aperture area, have been previously chosen. The resulting aperture area is 67.52 ${\text{m}}^{2}$. We optimize the optical efficiency for operation at 600 C∘, identifying the key parameters that influence performance. The system achieves a power output greater than 45 kW under direct radiation of 900 ${\text{W/m}}^{2}$. Additionally, maintaining optical efficiency above 74% requires a tracking error below $0.2^{\circ }$, with peak radiation values at the receiver reaching 26 ${\text{kW/m}}^{2}$. These results underscore the potential of Cassegrain concentrator designs to advance CSP technology, suggesting that they could significantly enhance this field.

## 1 Introduction

Concentrated Solar Power (CSP) technologies offer significant promise as a renewable energy source, mainly due to their ability to intermittently generate power when integrated with thermal storage systems [1]. A prominent example of a CSP tower plant that employs thermal storage is the Gemasolar facility, located in Seville. This plant uses molten salts, reaching temperatures of up to 600 C∘, and exhibits a capacity of 19.9 MWe [2]. Despite its potential benefits, conventional CSP tower plants involve the transport of thermal energy from the receiver to storage tanks through pipes, which implies significant infrastructure costs and thermal losses [3]. Furthermore, radiation losses that occur in the receiver can decrease the efficiency of the system, posing significant challenges.

To better understand the classification and different configurations of CSP technologies, readers are referred to a comprehensive review of these systems [4], where the technical construction and components of various CSP technologies are extensively discussed, with particular attention to Dish/Stirling concentrated solar power systems.

**Figure 1:**
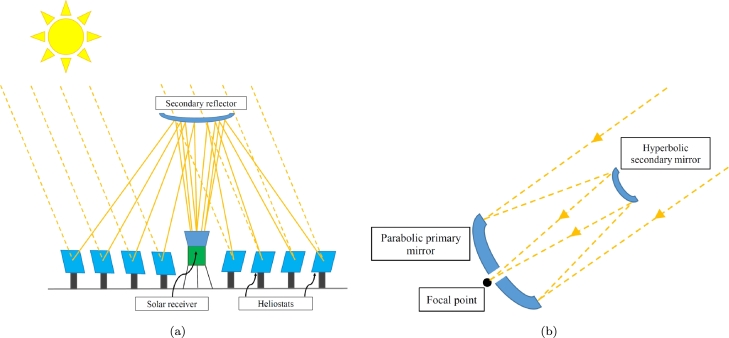
(a): Operating principle of a beam-down type central tower solar concentrator. (b): Optical principle of the Cassegrain telescope.

In recent years, the beam-down concept has gained attention as a potential solution to these problems [5]. This approach utilizes two-stage reflectors to concentrate solar radiation closer to the ground, resulting in a more compact system with reduced height and reduced maintenance requirements; see Fig. 1a. Furthermore, integrating a reactor or a thermal storage system closer to the receiver helps mitigate the disadvantages associated with conventional CSP technology. Some operating examples of central tower-type beam-down systems are located in Masdar (United Arab Emirates) [6], [7], in Miyazaki (Japan) [8], and in Italy [9].

Alternatively, solar dish collectors have recently gained significant attention in the field of CSP technology. This is due to their remarkable efficiency, effectiveness in terms of cost per kWh, when compared to parabolic trough systems, and their ability to serve as self-contained electric generators, making them suitable for power plants with a capacity ranging from 1 kW to several megawatts [10]. However, conventional designs have the disadvantage of placing the receiver above the primary mirror, which can increase complexity and maintenance costs. This issue becomes particularly relevant when developing technologies for thermal energy storage, as is the case with conventional tower plants, where the aforementioned advantages of beam-down technology still apply. In response to these challenges, beam-down technology on solar dish collectors is attracting significant attention [5].

In 1672, the physicist Laurent Cassegrain published in *Journal des sçavans* a new telescope design. The telescope was made up of a primary (parabolic) reflector that concentrates light at the focal point of a secondary (hyperbolic) reflector. This second reflector reflects the light towards the other focal point of the hyperbola to generate the image; see Fig. 1b. The Cassegrain design can be employed in many applications, such as antennas [11] and solar concentrators [12]. Using this principle, a beam-down solar dish collector can be designed. The first case of this technology was presented in 1979 by Mauk et al. [13], where a furnace was placed at the lower part of a paraboloid dish primary concentrator. An upper secondary hyperboloid reflector redirected the sun rays to the furnace.

Most studies on beam-down solar technologies focus on tower reflector systems and dish reflectors. Comparisons reveal that dish technologies boast higher optical efficiencies (78–87%) compared to tower systems (approximately 57%) [5]. This fact is associated with cosine effects [14].

Given the aforementioned factors, including the drawbacks associated with conventional CSP technologies, and the enhanced optical efficiency of beam-down dish systems, this paper introduces a Cassegrainian concentrator for thermal applications with a nominal power of 45 kW. Before detailing our design and its outcomes, we review previous studies to contextualize the novelty of our work.

O.J. Nydal [15] analyzed the optical design of a Cassegrainian solar concentrator for thermal applications using ray tracing software. The study focused on determining the effect of the position of the secondary reflector, sensitivity to tracking errors, and the effect of the size of the flat mirrors for the primary reflector (in addition to considering curved surfaces). All other geometrical parameters were kept fixed. Meng et al. [16] analyzed the optical design of a Cassegrainian solar concentrator with predetermined structural parameters using the TracePro software system. The purpose of the study was to investigate the effect of the solar angular size and angular error in mirror reflection. The proposed design consisted of a mixed photovoltaic-thermal (PVT) receiver where solar radiation that impacts the edges of the focal zone is used by PV cells for direct electricity production, while solar energy hitting its central region was used in a thermal receiver to produce heat. Dhäler et al. [17] conducted a comprehensive study on the optical design and experimental characterization of a Cassegrainian solar concentrator for fuel production through thermochemical redox cycles. They analyzed seven different configurations of the concentration system with fixed structural parameters, obtaining power as a function of the solar reactor's aperture radius. The system alternates two reactors intermittently. The optical analysis was carried out using the ray tracing software LightTools.

In the studies just referenced, most of the geometric parameters that define the optical system are determined prior to the optimization process and optical analysis of the system. This can cause that the optimal solutions found are not globally optimal for the proposed application, leaving room for improvement so that systems can achieve even higher efficiencies. In this regard, in a recent bibliographic study on double-stage solar concentrators conducted by Malik et al. [10] (most of which are of Cassegrainian type) it is concluded that most of the reviewed studies only validate the proposed systems experimentally, and that analytical results could help identify future improvements. In addition, parametric models can also help identify better designs, combining geometry and environmental conditions, according to energy applications. Recent studies have applied parametric analyses specifically to Solar Dish-Stirling Systems, demonstrating their usefulness in optimizing power output and efficiency [18], [19].

Furthermore, beam-down solar dish systems have certain limitations that need to be overcome in order to make them a mature technology. One of the main advantages of these systems is their reduced height, which can lead to more intense ray dispersion effects but requires the optical design of the system to be performed with high accuracy [5]. One limitation is the lower optical efficiency compared to conventional systems due to the incorporation of multiple reflecting surfaces. These limitations highlight the need for precise optical simulations that consider the real optics of the system using advanced methods. Ultimately, investigating these geometries is essential, as they offer the significant advantage of placing the heaviest equipment on the ground, simplifying installation and maintenance.

Building upon the aforementioned works, this article presents a comprehensive study focused on determining the optimal design of a Cassegrainian solar concentrator through optical simulation. Our investigation incorporates various aspects, including the consideration of variable design parameters, a realistic optical model for the materials, assessment of reflected dispersion, and the modelization of the size of the sun [20]. Optical simulations were carried out using the web tool OTSunWebApp [21], which is available at the url https://otsun.uib.es and its documentation can be found at https://otsun-uib.github.io/. OTSunWebApp is a companion tool of the OTSun ray tracing software [22]. In fact, we used an evolution of this webtool, with the same computation engine, but with a different interface and an architecture based in containers (using docker) that allowed us to parallelize the computations using a cluster of computers. We will shortly report on this evolution and make it available to the scientific community. Hereafter, whenever we refer to OTSunWebApp, we will be referring to this evolution.

OTSunWebApp uses the program FreeCAD [23] to design the physical model of the solar concentrator, and applies an accurate optical model using the Fresnel optical equations. The distinguishing aspect of our study lies in the development of a parametric model comprising six design parameters. This model aims to identify the most efficient design within predetermined structural constraints for its thermal application, targeting a power output of 45 kW at a working temperature of 600 C∘. Furthermore, using the software system OTSun has allowed us to work with CAD-type objects emulating the boundary and dimensions of each of the elements that constitute the system, applying an accurate model for the optics involved in real conditions [24]. The ultimate goal is to design a beam-down solar collector with a nominal design power of 45 kW. In this work, our focus is on the optical design, where radiation losses are considered. This analysis will provide the foundation for future research into the heat transfer, thermal energy storage capabilities, and power production potential of the solar concentrator.

## 2 Geometrical design of the Cassegrainian concentrator

**Figure 2:**
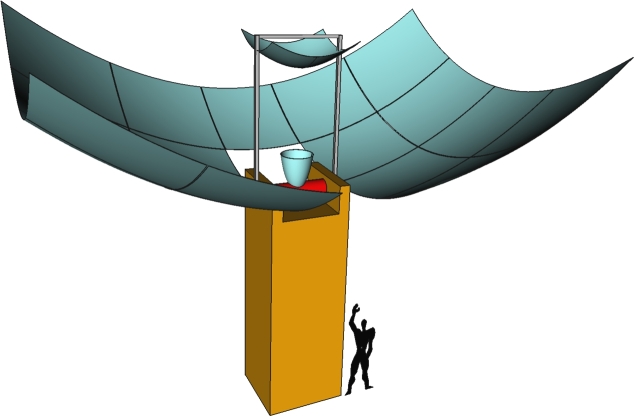
Experimental setup of the Cassegrain concentrator along with a Le Corbusier Module for reference of the dimensions.

**Figure 3:**
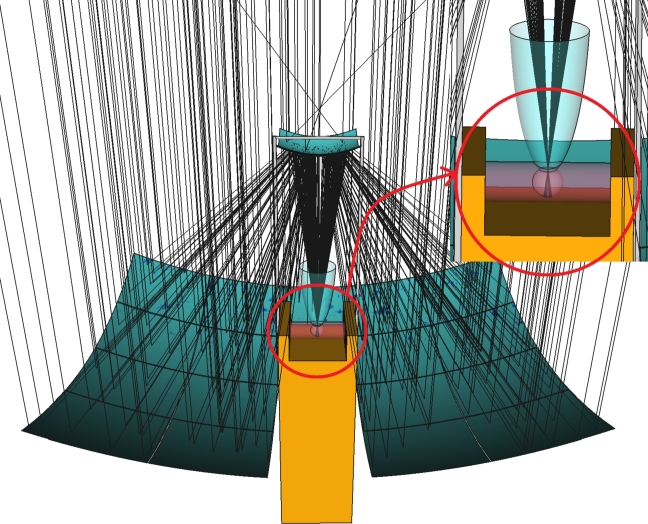
Optical path of solar rays under ideal conditions (assuming no optical errors in materials and considering the sun as a punctual source), illustrating the details of the absorber sphere, focal point, and the heat transfer mode through a cylindrical heat exchanger.

The design of the solar concentrator consists of a tower and a set of optical components. Within the tower, there are provisions for housing the receiver system, storage elements, and solar energy conversion devices. The optical components include a faceted primary parabolic mirror, a hyperbolic secondary mirror, and a compound paraboloid mirror connected to the receiver sphere. In addition, several structural components are essential to support these optical elements. An illustration of the design is depicted in Fig. 2, where a Le Corbusier Modulor has been added as a reference to represent the actual dimensions of the system. Fig. 3 shows a plot of some traced rays in an idealized scenario, assuming no optical material errors and modeling the sun as a punctual source. Initially, the rays are reflected by the primary paraboloid mirror towards the focal point of the secondary hyperboloid. Subsequently, the rays are directed towards the receiver sphere, where a compound paraboloid has been incorporated to enhance ray collection, which is relevant when both the sun cone and potential optical errors are considered (as discussed later on). Under ideal conditions, the focal point is located exactly at the center of the compound paraboloid, located at the circular aperture of the receiver sphere, as shown in Fig. 3. It is worth noting that certain elements in the illustration have been rendered with transparency to allow the visibility of the traced rays.

It should be noted that the integration of a spherical receiver offers substantial advantages, as exemplified in the study by E. Bellos [5] on the effectiveness of cavity receivers. This approach improves the concentration ratio, reduces thermal losses, and ensures a uniform heat flux distribution. Once solar radiation is captured by the receiver sphere, its temperature raises, and the heat is dissipated through convection via the working fluid circulating through the cylindrical heat exchanger depicted in Fig. 3. In this article, we focus on optical aspects and hence we do not address in detail the problem of modeling the transfer of heat, for which we use a simplified model to quantify radiative losses and their impact on power gain.

### 2.1 Structural model

**Figure 4:**
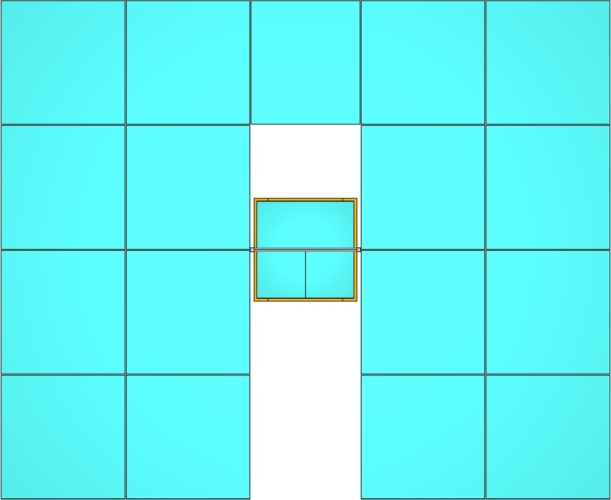
Top view of the solar concentrator, illustrating the overhead perspective.

**Figure 5:**
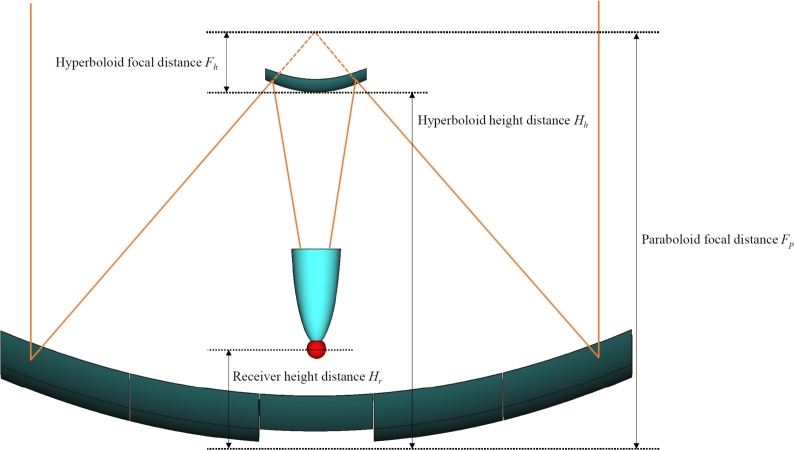
Sketch showing the locations and focal distances of the main elements, such as the hyperboloid and receiver placement, and focal distances of the primary and secondary mirrors. In orange, the image illustrates the optical path of a solar ray to showcase the ideal path.

The overall structure of the solar concentrator has already been shown in Fig. 2. Fig. 4 shows a top-down view of the design, and Fig. 5 gives the main optical parameters of the overall system. Note that it is not possible to add an extra mirror on the other side of the tower, since the overall system has to move in order to follow the sun. Indeed, the tower has to be mounted on a tracking system that allows it to rotate around its vertical axis, and the optical system can rotate around the horizontal axis to track the apparent movement of the sun; this last movement makes it impossible to place mirrors at the front side of the tower. The simulation of both movements can be modeled using the OTSunWebApp software, which computes the rotations that have to be applied to the system to track the sun. Since the results do not depend on the position of the sun, once we assume that the system is tracking the sun, we can consider the sun as located at the zenith.

The primary parabolic mirror is composed of seventeen sections: Two sets of eight mirrors each one, located at both sides of the tower, and one extra mirror between them. Each mirror is defined as the intersection of the paraboloid that has to be modeled, whose focal distance is $F_{p}$, with a 2m×2m square prism, except for the central mirror, where the basis of the prism is a 1.76m×2m rectangle. A distance of 20mm is maintained between the sections of the mirror to facilitate their positioning. With this configuration, the net aperture area of the primary reflector is 67.52 ${\text{m}}^{2}$. Finally, the central tower is a square prism with basis a square 1.66m×1.66m and with a height of 5m. There is a 70mm gap between the tower and the nearer mirrors, which facilitates the installation of structural support elements.

The hyperbolic secondary mirror, with square section, has its vertex at a distance $H_{h}$ from the vertex of the primary mirror (see Fig. 5) and has a focal distance $F_{h}$. Its dimensions are calculated to ensure the interception of all reflected rays. To make this computation, a reference ray is generated, originating from one of the corners of the primary reflector, and its path to the receiver is computed taking into account the optical deviations caused by the angular size of the sun, the specular dispersion of the primary mirror, the position error of the mirrors and the tracking error.

In the presence of multiple error sources, each characterized by a Gaussian distribution with a standard deviation $\sigma_{i}$, the resulting angular error can be represented by a distribution with a standard deviation $\sigma_{b}$ according to the Central Limit Theorem [25]:

(1) $$\sigma_{b}^{2}=\sum_{i=1}^{n}\sigma_{i}^{2}$$

The specular angle deviation of rays reflected by mirrors is characterized by the deviation $\sigma_{b}$ from Eq. (1) and is a combination of the following error sources: solar disk $\sigma_{SD}$, error due to non-specularity from microscopic effects $\sigma_{s1}$ (scattering dispersion), tilt error of the mirrors $\sigma_{t}$, mirror alignment error $\sigma_{a}$, and tracking mechanism error $\sigma_{tr}$. It is important to note that a factor of 2 must be included due to the law of reflection:

(2) $$\sigma_{b}^{2}={\left(2\sigma_{SD}\right)}^{2}+{\left(2\sigma_{s1}\right)}^{2}+{\left(2\sigma_{t}\right)}^{2}+{\left(2\sigma_{a}\right)}^{2}+{\left(2\sigma_{tr}\right)}^{2},$$

where $\sigma_{SD}=4.65\;\text{mrad}$ is the semi-angle of the solar disk, and $\sigma_{s1}=\frac{2.4529}{2}\;\text{mrad}$ is the specular standard deviation (as we expose latter). For the mirror positioning errors, we assume a value of 2 mrad for both tilt and alignment errors, $\sigma_{t}$ and $\sigma_{a}$ respectively. Regarding the tracking mechanism error, a tracking error of $0.2^{\circ }$ has been considered, corresponding to $\sigma_{tr}=3.49\;\text{mrad}$. Based on the combined error contributions, the total specular deviation is $\sigma_{b}=10.41\;\text{mrad}$.

The point of intersection of the rays with the hyperbolic surface is used to determine the boundary of the secondary reflector. Hence, the size of the secondary reflector depends on the design parameters that define the concentrator. Finally, Fig. 5 also illustrates the receiver height distance $H_{r}$, which is defined as the distance between the vertex of the primary mirror and the center of the receiving sphere.

**Figure 6:**
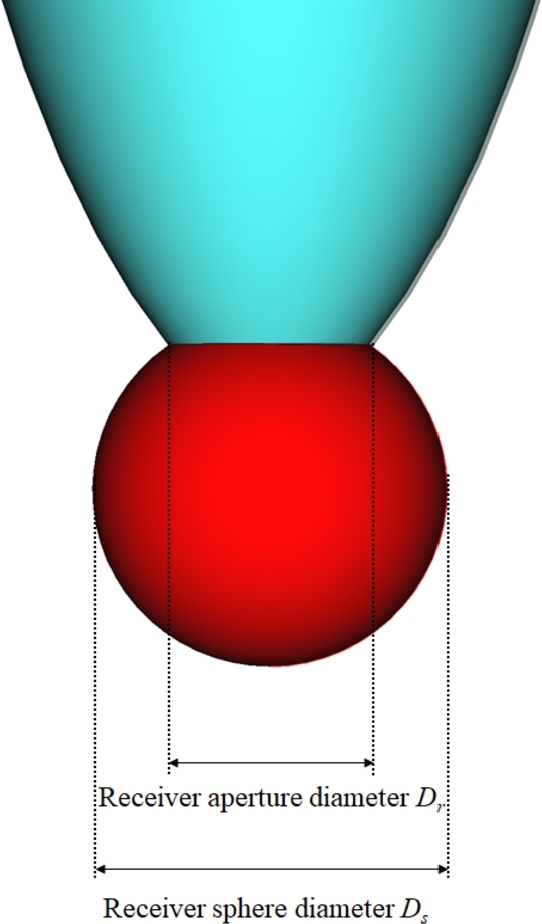
Parameters defining the absorber: receiver aperture diameter *D*_*r*_, and receiver sphere diameter *D*_*s*_.

**Figure 7:**
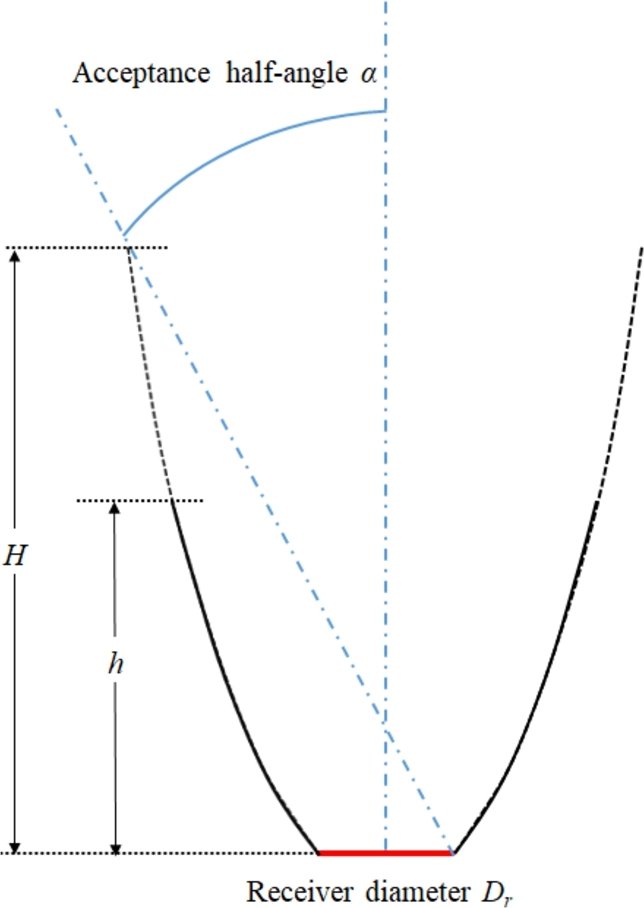
Visual representation of the geometric parameters of the compound paraboloid concentrator coupled to the receiver.

As for the receiver system, it consists of a sphere with diameter $D_{s}=300\;\text{mm}$ coupled to a compound paraboloid reflector. The parameter $D_{r}$ is the diameter of the circle defined by the intersection of the sphere with the paraboloid; see Fig. 6 for a depiction. Note that adding a third mirror has the inconvenience that more reflections take place. However, it is compensated by the increase of the interception factor. Regarding the compound paraboloid geometry, it is determined by the acceptance half-angle *α*, and the truncation factor $\tau =\frac{H}{h}$, defined as the ratio between the theoretical height of the compound parabolic mirror, and real height considered. See Fig. 7 for a depiction of these parameters.

In summary, there are six geometric parameters that structurally define the solar concentrator: the paraboloid focal distance $F_{p}$, the hyperboloid focal distance $F_{h}$, the receiver height distance $H_{r}$, the receiver aperture diameter $D_{r}$, the acceptance half-angle *α* and the truncation factor *τ*. These parameters and other technical details are summarized in Table 1.

**Table 1:** Technical specifications and dimensions of the Cassegrainian concentrator system.

Component	Specification	Detail
Primary mirror	Number of sections	17
	Mirror size	Two sets of 8 mirrors each, plus 1 extra mirror
	Distance between sections	20 mm
	Net aperture area	67.52 m^2^
	Focal distance	*F*_*p*_ (variable parameter)
Secondary mirror	Focal distance	*F*_*h*_ (variable parameter)
	Size	Calculated to ensure interception of all rays
Receiver sphere	Diameter	300 mm
	Receiver height distance	*H*_*r*_ (variable parameter)
CPC Reflector	Aperture diameter	*D*_*r*_ (variable parameter)
	CPC acceptance half-angle	*α* (variable parameter)
	CPC truncation factor	*τ* (variable parameter)
Central tower	Dimensions	Square prism with base of 1.66 m × 1.66 m and height of 5 m

### 2.2 Optical model

As already mentioned, in this study we have used an evolution of the webtool OTSunWebApp [21] that uses the same computation engine, based on the OTSun Python package [22], which allows the user to analyze solar harvesting devices regardless of its application (thermal/PV). In comparison to other ray tracing programs, such as the well-established Tonatiuh [26] and SolTrace [27], the tools based in OTSun take advantage of its direct implementation of the Fresnel optical equations, among other features. OTSun considers multiple optical characteristics, including wavelength-dependent light, solar angular size, specular reflectivity dispersion, optical interference from thin films, and the wavelength-dependent Lambert-Beer law for energy absorption when rays pass through materials, whether they are transparent or semiconductors. OTSun uses FreeCAD [23], an open-source parametric 3D modeling tool, for constructing the geometries of the solar harvesting devices, and for computing how these objects interact with the simulated rays. It is also worth mentioning that FreeCAD includes a Workbench called Optics, which enables solar concentrator simulations within this environment [28]. In this study, however, we rely on the OTSun environment, which has been validated in different settings [29], [30], [31], [22].

We provide now a description of the optical materials we have used for our simulation of the Cassegrainian concentrators.

#### 2.2.1 Mirror

The selected material for the solar mirrors is a first-surface reflector primarily composed of aluminum as the reflective layer, with an additional coating layer applied for protection. It is chosen for its high reflectivity and durability. Of the three mirrors in the solar concentrator, only the CPC mirror is subjected to elevated temperatures near the focal point. In such scenarios, heat dissipation occurs through natural convection with ambient air; however, if temperatures were to exceed acceptable limits, the reflective material could degrade, compromising performance. In this case, additional thermal management strategies, such as heat sinks, could be employed to mitigate the effects.

**Figure 8:**
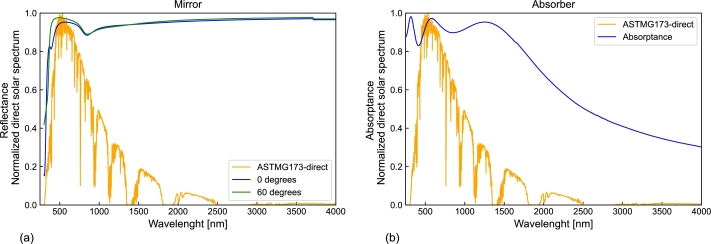
Spectral behavior of materials used in simulations, with the solar spectrum as a reference: (a) Reflectance of the mirror, (b) Absorptance of the receiver material.

We determined the optical properties of the chosen material using the results of Sutter et al. [32], [33], who previously investigated the optical characteristics and thin-film structure of these mirrors. We computed the spectral reflectance of this material using the tmm Python package developed by Steven Byrnes [34]. This package allows for the simulation of light propagation in planar multilayer thin films, considering the impact of multiple internal reflections and interference, using the Transfer Matrix Method (TMM) for the computations. See [32] for details of the coating stack. Table 2 gives, for each material, the thickness of the layer and a reference to its optical constants, obtained from the refractiveindex.info database of optical constants [35], and used to compute the refractive index. These values are essential for constructing the optical material within the OTSun system. Fig. 8(a) shows the spectral reflectance of the solar mirror for angles of incidence equal to $0^{\circ }$ (i.e. normal incidence) and $60^{\circ }$, where the direct solar standard spectrum AM1.5, according to ASTM G-173-03 [36], is provided as a reference for the solar spectrum.

**Table 2:** Thin layers of the first-surface mirror showing materials, thicknesses, and sources for refractive index.

Material	Thickness [nm]	Reference
SiO_2_	3000	[37]
TiO_2_	60	[38]
SiO_2_	95	[37]
Al (pure)	65	[39]
Al (substrate)	–	[39]

Additionally, in our model, the specular scattering behavior of the reflector material was characterized using the results by Sutter et al. [33], where the specular reflectance is given by

(3) $$\rho (\lambda ,\theta_{i},\varphi )=\rho (\lambda ,\theta_{i},h)[1-ke^{-\frac{\varphi^{2}}{2\sigma_{1}^{2}}}-(1-k)e^{-\frac{\varphi^{2}}{2\sigma_{2}^{2}}}],$$

where ρ(λ,θ,h) is the hemisferical reflectance at wavelength *λ* and at an incidence angle $\theta_{i}$; $\sigma_{1}$ and $\sigma_{2}$ are the statistical standard deviations; *k* is a weight parameter; and *φ* is the acceptance angle (half-space).

It is important to note that in the implementation of the scattering behavior, the standard deviations to be used should be those obtained from experiments, divided by two. This adjustment is necessary because, in the OTSun framework, the deviation of the surface normal vector is considered, whereas Sutter et al. [33] focus on the deviation of the reflected ray.

For additional details regarding these parameters, we direct the reader to the OTSunWebApp materials manual, available at https://otsun-uib.github.io/manuals/materials.pdf. In this study, we used materials that exhibit deviations from specular reflection, employing a method based on the deviation of the normal to the surface of the reflector. Taking into account all the factors mentioned above, the standard deviations for the normal vector of the reflector surface in our simulations are $\sigma_{s1}=\frac{2.4529}{2},\text{mrad}$, $\sigma_{s2}=\frac{16.0024}{2},\text{mrad}$, and k=0.9226.

#### 2.2.2 Absorber

The absorber material selection is crucial and depends on the operating conditions of the device. Mo-Si-N-based semiconductor-ceramic hybrid materials are an appropriate option for high-temperature and high-pressure environments due to their excellent properties, including high resistance to oxidation, high thermal conductivity, and low emissivity in the infrared region [40]. The material used for the absorber sphere in our model was the one considered by Rodriguez-Palomo et al. in [41], where the thermal stability of solar selective coatings based on the novel MoSi_2_-Si_3_N_4_ hybrid composite material was investigated. Fig. 8(b) shows the spectral reflectance of this material at a working temperature of 600 C∘ after 515 hours, where the emissivity is 0.33±0.05 [41]. Fig. 8(b) displays the spectral absorptance of the receiver material, along with the direct solar standard spectrum AM1.5 according to ASTM G-173-03 [36], as a reference.

## 3 Methods and results

He has used a multi-step methodology to optimize the optical performance and energy balance of the system. It encompasses optical simulations with varying parameters to eliminate non-optimal configurations, analysis of optical efficiency to identify optimal parameters, power balance calculations accounting for incident radiation and thermal losses, and a detailed evaluation of radiation distribution. In addition, it includes an error analysis of the accuracy of the tracking to assess its impact on efficiency. This structured approach enables a comprehensive understanding of the system's performance under realistic conditions. Finally, in line with the principles of open science, the code used to generate the geometries, the ray tracing results via OTSunWebApp, and the scripts for generating the curves are publicly available at https://github.com/otsun-uib/CassegrainSuppMat.

### 3.1 Optical efficiency

Once the structural design and optical model were established, the next step was to define the specific geometric parameters to construct the Cassegrain designs. Two sets of simulations were performed to identify the optimal configurations. The first set, simulating the emission of 50,000 rays per case, aimed to dismiss parameter values that were clearly not optimal. Subsequently, a second set of simulations was performed with the emission of 200,000 rays per case to enhance the accuracy of the ray tracing results, thereby identifying the optimal optical designs (see below for details). The estimated error in our computation of the optical efficiency is ±1% and ±0.5% for 50,000 and 200,000 rays, respectively, with a confidence level of 98%, as illustrated in Appendix A.

The simulations, as previously noted, were done with an evolution of OTSunWebApp. In particular, using the computation named *Total Analysis (AM1.5 ASTM G-173-03 Direct)* therein, which models the optical response of the solar concentrator considering the direct solar spectrum ASTM G-173-03 [36], with an irradiance of 900 ${\text{W/m}}^{2}$. The parameters used in these computations, as needed to run the simulation, are given in Table 3.

**Table 3:** Summary of input parameters for simulating Cassegrain designs using the *Total analysis (AM1.5 ASTM G-173-03 direct)* mode in OTSunWebApp.

Input name (as it appears in OTSunWebApp)	Value
Number of rays	50,000/200,000
Aperture collector for PV [mm]	Empty
Aperture collector for thermal [mm]	67520000
Ray distribution from the source (CSR value)	0.05

Table 4 lists the parameters used for the initial set of designs, giving their corresponding symbols and the range of values (in the form of: start, last, step) that were used. This provided the foundation for generating 17,280 FreeCAD files. However, some were discarded due to impractically short distances (<100mm) between the CPC and the secondary reflector, or because the semi-major angle of the hyperbola was negative, which is physically impossible. The remaining 13,549 files were analyzed in the first round of ray tracing simulations.

**Table 4:** Geometric design parameters, their corresponding symbols, and the ranges of values (initial, final, step) used for the first round of simulations.

Name	Symbol	Initial	Final	Step
Paraboloid focal distance [mm]	*F*_*p*_	1000	4500	500
Hyperboloid focal distance [mm]	*F*_*h*_	100	900	200
Receiver height distance [mm]	*H*_*r*_	−400	600	200
Receiver aperture diameter [mm]	*D*_*r*_	200	240	20
CPC acceptance half-angle [^∘^]	*α*	14	24	2
CPC truncation factor	*τ*	0.2	0.8	0.2

**Figure 9:**
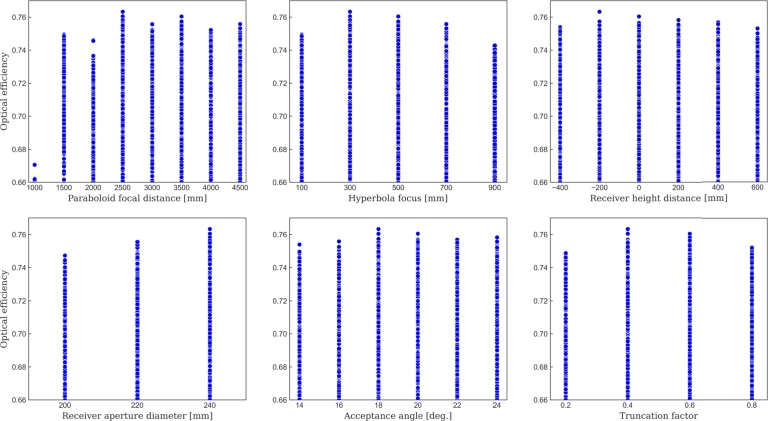
Optical efficiencies for the first set of designs, based on the parameters detailed in Table 4. Only the cases with efficiency greater than or equal to 66% are displayed. Parameter analysis includes: Paraboloid focal distance, hyperboloid focal distance, receiver height distance, receiver aperture diameter, acceptance angle, and truncation factor.

Fig. 9 displays the results obtained for the optical efficiency depending on the different parameters, and where the cases with an efficiency lower than 66% have been discarded to ease the visualization of the most relevant cases. From the observation of these plots some remarks arise. First, the paraboloid focal distance, $F_{p}$, exhibits interesting values clustered around 2500 mm. However, no clear trend is observed in relation to the values that offer higher optical efficiency. Therefore, for a comprehensive understanding, it is advisable to explore the range of values centered precisely at 2500 mm with greater precision. Regarding the hyperboloid focus distance, $F_{h}$, the highest efficiency is observed at 300 mm, and therefore, further analysis should focus on values close to this distance. Regarding the receiver height distance, $H_{r}$, optimal values are concentrated between -200 mm and slightly higher values. Hence, it is crucial to explore the optical performance in the range from -200 mm to 400 mm.

When examining the aperture diameter of the receiver, $D_{r}$, a positive correlation with efficiency is observed as the value increases, but preliminary internal investigations have indicated that surpassing a diameter of 240 mm is not advisable due to the resulting reduction in the heat exchange surface area of the absorber sphere with the surroundings, and hence the upper limit for this parameter is set at 240 mm. Regarding the acceptance angle *α*, optimal values are mainly centered around $18^{\circ }$. Although there is no need to expand the range of values in further simulations, a closer examination of the behavior near this critical value is warranted. Lastly, the truncation factor, *τ*, reveals optimal values precisely at midpoints of the considered range, specifically at 0.4 and 0.6. Hence, a more detailed analysis of values centered around 0.5 is required.

Based on these observations, the analysis was extended to a second set of 20,580 files, which were reduced to 19,709 after discarding those that resulted in impractical designs, as in the first round. This set aimed to explore further the ranges of interest for the parameters, taking lower values for the steps and increasing the number of emitted rays in each simulation to 200,000. The values of the parameters used in the simulation are shown in Table 5.

**Table 5:** Geometric design parameters, their corresponding symbols, and the defined ranges of values (initial, final, step) for the second series of simulations.

Name	Symbol	Initial	Final	Step
Paraboloid focal distance [mm]	*F*_*p*_	1500	4500	500
Hyperboloid focal distance [mm]	*F*_*h*_	100	600	100
Receiver height distance [mm]	*H*_*r*_	−200	400	100
Receiver aperture diameter [mm]	*D*_*r*_	220	240	20
CPC acceptance half-angle [^∘^]	*α*	17	23	1
CPC truncation factor	*τ*	0.3	0.7	0.1

**Figure 10:**
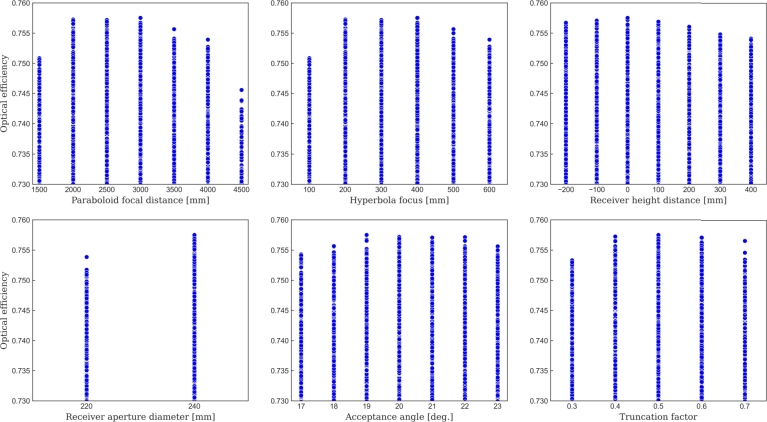
Optical efficiencies for the second set of designs, based on the parameters detailed in Table 5. Only cases with efficiency greater than or equal to 73% are displayed. Parameters analyzed are: Paraboloid focal distance, hyperbola focal distance, receiver height, receiver aperture diameter, acceptance angle, and truncation factor.

Fig. 10, displays the computed optical efficiencies for the second set of parameters. Based on these results, optimal cases can be identified for all parameters. The design with the maximum efficiency corresponds to the following choice of parameters: $F_{p}=3000\;\text{mm}$, $F_{h}=400\;\text{mm}$, $H_{r}=0\;\text{mm}$, $D_{r}=240\;\text{mm}$, $\alpha =19^{\circ }$, and τ=0.5. This design achieves an optical efficiency of 75.75%.

### 3.2 Power balance

After the determination of the geometries optimizing the optical efficiency, a comprehensive power balance analysis was performed to find their nominal power. This analysis took into account an incident solar radiation of 900 ${\text{W/m}}^{2}$ as well as radiative losses from the receiver operating at 600 C∘. To quantify these losses, a model based on Planck's blackbody radiation was employed, utilizing the Stefan-Boltzmann law:

(4) $$P_{r}=\epsilon \sigma A_{r}T^{4}.$$

In this equation, $P_{r}$ represents the total radiative heat power emitted by the receiver, *σ* is the Stefan-Boltzmann constant ($5.67\times 10^{-8}\;\text{W}\;{\text{m}}^{-2}\;{\text{K}}^{-4}$), $A_{r}=\frac{\pi D_{r}^{2}}{4}$ denotes the aperture area of the absorber sphere, *T* is the working temperature (873.15 K), and ϵ=0.33 is the emissivity of the material. The material selected for the absorber sphere is a MoSi_2_-Si_3_N_4_ hybrid composite [41], as previously mentioned.

The power balance equation that gives the useful power absorbed by the sphere is

(5) $$P_{u}=\eta_{\text{opt}}G_{\text{DNI}}A-P_{r}.$$

Here, $\eta_{\text{opt}}$ represents the optical efficiency of the system, $G_{\text{DNI}}=900\;{\text{W/m}}^{2}$ denotes the incident direct normal irradiance, and *A* stands for the aperture area of the solar concentrator, $67.52\;{\text{m}}^{2}$. The term $P_{r}$ accounts for the radiative losses, given by Equation (4).

In Table 6 we present the ten cases with greater power balance, giving both the design parameters and the corresponding useful power ($P_{u}$) and optical efficiency ($\eta_{opt}$) for each of them. These optimal designs demonstrate a generated power ranging from 45.48 to 45.54 kW and an optical efficiency between 75.66% and 75.75%. Notably, the most optically efficient designs also exhibit higher absorbed power, despite accounting for thermal losses due to radiation at the receiver. These findings provide valuable insight into the specific values of the parameters that contribute to the efficiency of the presented Cassegrain solar concentrator design. The solar concentrator illustrated in Fig. 2 corresponds to the most efficient case listed in Table 6.

**Table 6:** Results for the ten designs with the highest absorbed power by the receiver.

*P*_*u*_ [kW]	*η*_*opt*_[%]	*F*_*p*_ [mm]	*F*_*h*_ [mm]	*H*_*r*_ [mm]	*D*_*r*_ [mm]	*α* [^∘^]	*τ*
45.54	75.75	3000	400	0	240	19	0.5
45.53	75.73	2000	200	0	240	20	0.4
45.52	75.72	2500	300	0	240	20	0.5
45.52	75.71	2500	300	0	240	22	0.6
45.51	75.71	2000	200	−100	240	21	0.6
45.50	75.69	2500	300	100	240	20	0.5
45.49	75.67	3000	400	−200	240	19	0.5
45.49	75.66	2500	300	−200	240	20	0.5
45.49	75.66	2500	300	100	240	19	0.4
45.48	75.66	2000	200	−100	240	19	0.5

### 3.3 Tracking error

The deviation in the position of the sun, which represents the tracking error, is a critical factor in analyzing the performance of high solar concentrating systems. Given the optimal designs identified from the results presented in Table 6, which are ranked from highest to lowest power absorption, two designs deserve special attention. These designs, referred to as Case 1 and Case 2, occupy the first two positions in the table. Notably, although both designs demonstrate similar absorbed power, they differ significantly in their design parameters. Specifically, Case 2 exhibits greater optical compactness compared to Case 1, with the vertex of the secondary mirror positioned 800 mm closer.

**Figure 11:**
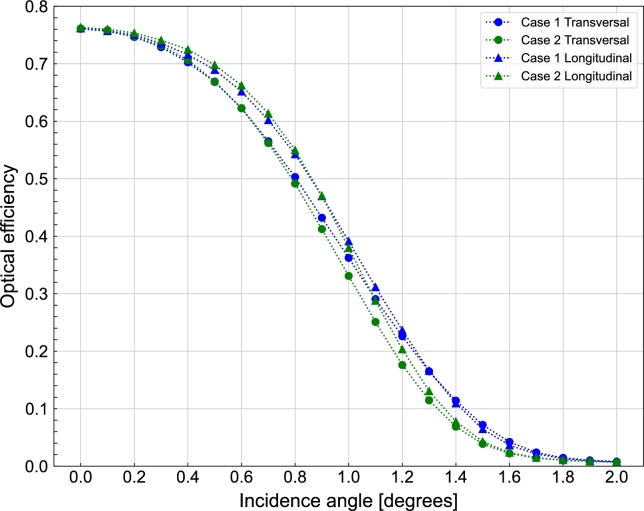
Comparison of optical efficiency between Case 1 and Case 2 in transversal and longitudinal planes, with the solar angle deviation representing the tracking error of the system.

Using these two designs as reference points, the optical efficiency of both designs is analyzed when the position of the sun deviates by a certain angle, ranging from $0^{\circ }$ to $2^{\circ }$, in both the transversal and longitudinal planes. The longitudinal plane is defined as the plane that aligns with the axis of the tower gaps. As shown in Fig. 11, the optical efficiency in the transversal plane exhibits greater sensitivity to tracking errors compared to the longitudinal plane. Finally, regarding the tracking error, to maintain an efficiency above 74% in both cases, it is necessary to have a tracking system with a precision of $0.2^{\circ }$, as illustrated in Fig. 11.

### 3.4 Radiation distribution on the receiver

Understanding the distribution of concentrated solar radiation on the receiver is essential for analyzing the conversion efficiency of solar radiation into heat. To achieve this, a *Spectral Analysis (single solar direction)* simulation was conducted using the OTSunWebApp tool, which allows users to determine the optical response for a specified range of wavelengths. This simulation provides detailed information about each ray that impacts the receiving sphere, enabling quantification of radiation flux. The parameters used for this simulation are listed in Table 7. A total of 5,000 rays were emitted for each wavelength, ranging from 280 nm to 3000 nm, at 10 nm intervals. This methodology ensures comprehensive coverage of the relevant solar spectrum and facilitates precise analysis of the radiation distribution on the receiver.

**Table 7:** Summary of inputs for the simulation of Case 1 and Case 2 using *Spectral Analysis (single solar direction)* in OTSunWebApp.

Input name (as it appears in OTSunWebApp)	Value
*ϕ*_*sun*_ (solar azimuth angle) [°]	0
*θ*_*sun*_ (solar zenith angle) [°]	0
Wavelength Initial [nm]	280
Wavelength Final [nm]	3000
Wavelength Step [nm]	10
Number of rays	5000
Aperture collector for PV [mm^2^]	–
Internal Quantum Efficiency	–
Aperture collector for thermal [mm^2^]	67520000
Ray distribution from the source (CSR value)	0.05

**Figure 12:**
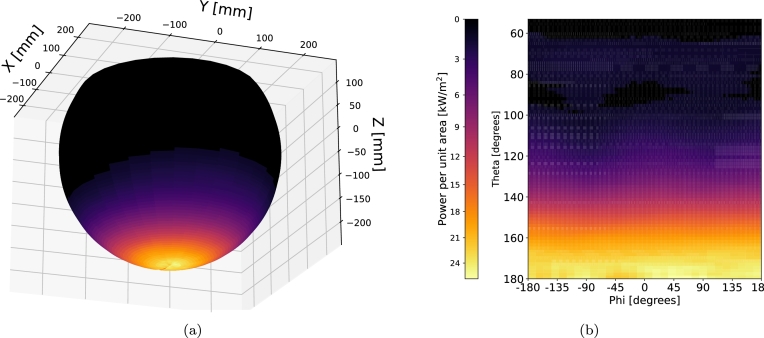
Radiation flux distribution for Case 1: (a) Heat map in spherical coordinates, (b) 3D representation of the absorbing sphere.

**Figure 13:**
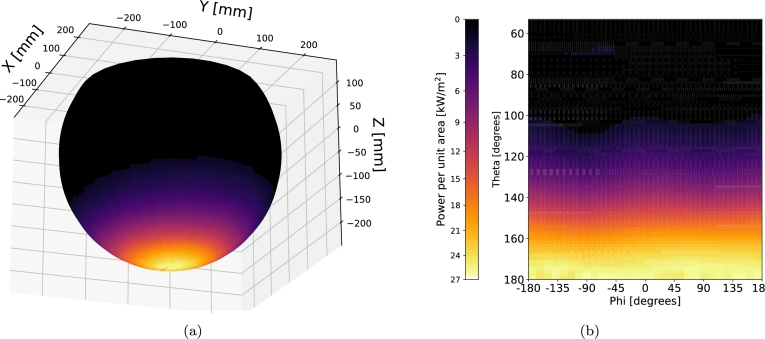
Radiation flux distribution for Case 2: (a) Heat map in spherical coordinates, (b) 3D representation of the absorbing sphere.

Figure 12, Figure 13 illustrate the radiation distribution on the absorbing sphere for Cases 1 and 2, respectively, from Table 6. In Fig. 12a, the radiation flux within the sphere is depicted in a 3D representation for Case 1. To provide a more detailed insight into the radiation distribution, Fig. 12b presents a heat map plotted using spherical coordinates. Here, the angle *θ* corresponds to the zenith angle, while *ϕ* represents the azimuthal angle, both taken from the center of the sphere. The visualization highlights that the maximum radiation is concentrated in the lower parts of the sphere and remains relatively consistent across different azimuthal angles. The peak radiation intensity is recorded at 25.9 ${\text{kW/m}}^{2}$. Figs. 13a and 13b show analogous representations for Case 2. Here, a uniformly distributed radiation pattern is observed with respect to the angle *ϕ*, reaching a maximum radiation intensity of 26.6 ${\text{kW/m}}^{2}$ at the lower portion of the sphere, mirroring the findings observed in Case 1.

### 3.5 Comparative discussion of other beam-down dish systems

In our study, the optimal geometry achieved a simulated optical efficiency of 75.7%, assuming realistic conditions such as the spectral behavior of materials, scattering in reflective surfaces, the angular size of the sun using a CSR (circumsolar ratio) of 0.05, and radiation losses at the receiver. Although comparisons are complex due to the different assumptions made in each study, several conclusions can be drawn when comparing with other parabolic dish beam-down systems.

For instance, Yang et al. [42] reported a peak optical efficiency of 77.8%. However, their optical model does not account for critical factors such as scattering in the reflectors, mirror reflectivity, or the angular size of the sun, resulting in an overestimated efficiency under real-world conditions. Similarly, Xu et al. [43] reported a peak efficiency of 79%, but their model also did not consider specular scattering or the angular size of the sun. Both cases present idealized models, which would likely yield lower efficiencies under more realistic assumptions as those we have made.

On the other hand, Yang et al. [44] reported a peak optical efficiency of 73%, using Buie's model to account for the sun's angular size (CSR = 0.05), with mirror reflectivity assumed at 0.935 and a slope error of 1.3 mrad. This study is directly comparable to ours, as it employs a similarly realistic approach. Our optical efficiency of 75.7% aligns closely with their results, with minor differences likely attributable to variations in the concentrator geometry or material properties.

Finally, Dahler et al. [17] reported an experimental peak optical efficiency of 60%. This lower efficiency is expected in experimental settings due to practical factors such as construction imperfections and environmental conditions, which typically reduce performance compared to simulations.

In summary, although direct comparisons with other studies are challenging due to the different assumptions in the models and variations in design parameters, our results are well aligned with those using realistic models, such as Yang et al. [44], and show better performance than idealized models that overlook key factors.

## 4 Conclusions

The Cassegrain-type solar concentrator, with its beam-down configuration, emerges as a strong candidate for addressing the challenges inherent in CSP technology, particularly those related to radiation losses at the receiver and heat transport through pipes. In this sense, optimizing complex geometries is crucial; hence, optical simulations arranged with parametric models are essential for optimizing the efficiency of the design.

This study analyzed a Cassegrain-type solar concentrator with a 67.52 ${\text{m}}^{2}$ aperture surface, parameterized by six geometric parameters, using OTSunWebApp. This tool integrates ray tracing with CAD-based design and considers the wavelength dependence of optical materials. Material selection plays a strategic role; hence, a first-surface reflective aluminum material was chosen for the mirrors, and the absorber was made of a Mo-Si-N-based semiconductor-ceramic hybrid material, for which we considered their optical response as dependent of wavelength.

Two sets of simulations were conducted to explore the possible values for the design parameters. The first set involved emitting 50,000 rays for each of 13,549 designs, identifying the values to be discarded due to low optical efficiency. The second set involved varying the set of parameters under consideration, giving rise to the construction of 19,709 design cases, and increasing the number of rays to 200,000 to enhance the precision of the results. Additionally, considering a working temperature of 600 C∘, the power balance in the receiver was quantified, accounting for radiation losses and identifying the values giving the most efficient design.

Selecting the two most efficient cases, the results indicate that these designs can achieve a power output exceeding 45.50 kW under direct radiation of 900 ${\text{W/m}}^{2}$. Additionally, the sensitivity of the tracking mechanism was determined, revealing that a precision of $0.2^{\circ }$ is necessary to maintain optical efficiency above 74%. Furthermore, the spatial distribution of radiation within the absorber sphere shows azimuthal uniformity with a noticeable concentration in the lower regions, achieving peak values of 26 ${\text{kW/m}}^{2}$.

When comparing our findings with other parabolic dish beam-down systems, we find that we achieved a simulated optical efficiency of 75.7%, which is competitive with existing studies. While Yang et al. [42] and Xu et al. [43] reported higher efficiencies (77.8% and 79%, respectively), but their models overlooked critical factors like scattering and the angular size of the sun, making our results more realistic for practical applications. Furthermore, our efficiency is closely aligned with Yang et al. [44], who reported 73% under similar conditions, underscoring the robustness of our design approach. These findings suggest that optical models based on realistic models, such as those implemented in OTSunWebApp, allow for a more accurate understanding of the optical behavior of solar concentrators.

These findings highlight the potential of Cassegrain concentrator designs to advance CSP technology and justify further exploration into their practical implementation. Future work will focus on studying the optimal method for transferring heat to the working fluid, as well as analyzing the most efficient processes for both storage and direct conversion to electricity. Notably, the solar concentrator tower has been designed with dimensions that accommodate the necessary elements for these processes. Additionally, the structural elements to withstand mechanical loads and the tracking system's accuracy, as established in this study, will be subjects of further investigation.

## CRediT authorship contribution statement

**Ramón Pujol-Nadal:** Writing – review & editing, Writing – original draft, Validation, Software, Methodology, Investigation, Funding acquisition, Formal analysis, Data curation, Conceptualization. **Luis Guerreiro:** Supervision, Resources, Project administration, Investigation, Funding acquisition, Formal analysis, Conceptualization. **Gabriel Cardona:** Writing – review & editing, Supervision, Software, Investigation, Funding acquisition, Formal analysis.

## Declaration of Competing Interest

The authors declare that they have no known competing financial interests or personal relationships that could have appeared to influence the work reported in this paper.

## Figures and Tables

**Figure A.14:**
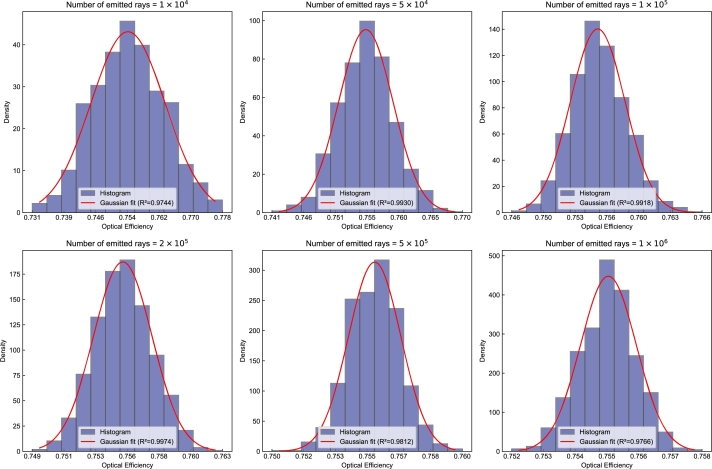
Histograms of optical efficiency for varying numbers of rays, relative to Case 1 in Table 6.

**Table A.8:** Parameters from Gaussian fits of optical efficiency distributions for various numbers of emitted rays, including the standard deviation of the data values for comparison with the obtained *σ* value.

Number of Rays	*μ*	*σ*	*σ*_data_
1 × 10^4^	0.7545	0.0093	0.0088
5 × 10^4^	0.7553	0.0042	0.0042
1 × 10^5^	0.7553	0.0028	0.0028
2 × 10^5^	0.7553	0.0021	0.0021
5 × 10^5^	0.7554	0.0013	0.0013
1 × 10^6^	0.7555	0.0009	0.0009

**Table A.9:** Error Intervals at different confidence levels.

Number of Rays	*δ* (95%)	*δ* (98%)	*δ* (99.75%)
1 × 10^4^	±1.7%	±2.0%	±2.7%
5 × 10^4^	±0.8%	±1.0%	±1.3%
1 × 10^5^	±0.6%	±0.7%	±0.9%
2 × 10^5^	±0.4%	±0.5%	±0.6%
5 × 10^5^	±0.2%	±0.3%	±0.4%
1 × 10^6^	±0.2%	±0.2%	±0.3%

## Data Availability

Data underlying the results presented in this paper can be found at the GitHub repository https://github.com/otsun-uib/CassegrainSuppMat.

## Appendix A Error in optical efficiency as a function of emitted rays

In this appendix we quantify the error committed in the computation of the efficiency using the OTSunWebApp ray tracing program, emphasizing the importance of accurately characterizing the system and understanding the error attributable to the number of emitted rays. To achieve this goal, we selected the geometry corresponding to Case 1 in Table 6 and performed simulations where the number of emitted rays were $1\times 10^{4}$, $5\times 10^{4}$, $1\times 10^{5}$, $2\times 10^{5}$, $5\times 10^{5}$, and $1\times 10^{6}$, respectively, and each simulation was repeated 1000 times to ensure statistical robustness.

The first step in determining the simulation error for optical efficiency was to verify if the distribution of results followed a normal distribution. This verification is illustrated in Fig. A.14, which presents histograms of optical efficiency for each simulation scenario, along with the best-fitting Gaussian probability distribution curve. It's important to note that the histograms presented in Fig. A.14 are normalized, so that they represent the probability density. Additionally, each graph shows the R-squared coefficient (coefficient of determination) of the fitted Gaussian, which ranges between 0.977 and 0.997 for all cases. This high R-squared value validates that the optical efficiency obtained with the OTSunWebApp program fits well to a normal distribution, indicating symmetry around the mean and predictable probabilities within standard deviations.

Upon analyzing the histograms, it was observed that the distribution of optical efficiency values tended to converge as the number of emitted rays increased, as expected. Table A.8 below presents key parameters extracted from the Gaussian fits of the optical efficiency distributions for each simulation scenario. These parameters include the mean (*μ*), Gaussian fit standard deviation (*σ*), and standard deviation from data ($\sigma_{\text{data}}$).

The robust fit to a normal distribution in the OTSunWebApp results validates the reliability of using standard deviation values from the original data ($\sigma_{\text{data}}$) to quantify errors in the optical efficiency obtained by the app. Under this assumption, an estimation of the error with a given a certain confidence level can be computed as

$$\delta_{CL}=Z_{\text{critical}}\times \sigma_{\text{data}},$$

where the critical value $Z_{\text{critical}}$ can be computed by the expression

$$Z_{\text{critical}}=\Phi^{-1}\left(1-\frac{1-CL}{2}\right),$$

where $\Phi^{-1}$ is the inverse function of the standard normal cumulative distribution and *CL* is the desired Confidence Level. Table A.9 gives the error estimations for the simulated optical efficiencies with different confidence levels, computed using the formula above.
